# Topical Alginate Protection against Pepsin-Mediated Esophageal Damage: E-Cadherin Proteolysis and Matrix Metalloproteinase Induction

**DOI:** 10.3390/ijms24097932

**Published:** 2023-04-27

**Authors:** Tina L. Samuels, Simon Blaine-Sauer, Ke Yan, Kate Plehhova, Cathal Coyle, Nikki Johnston

**Affiliations:** 1 Department of Otolaryngology and Communication Sciences, Medical College of Wisconsin, Milwaukee, WI 53226, USA; 2 Department of Pediatrics Quantitative Health Sciences, Medical College of Wisconsin, Milwaukee, WI 53226, USA; 3 Reckitt Benckiser, Hull HU8 7DS, UK; 4 Department of Microbiology and Immunology, Medical College of Wisconsin, Milwaukee, WI 53226, USA

**Keywords:** Alginate, pepsin, gastroesophageal reflux disease

## Abstract

Epithelial barrier dysfunction is a hallmark of gastroesophageal reflux disease (GERD) related to symptom origination, inflammatory remodeling and carcinogenesis. Alginate-based antireflux medications were previously shown to topically protect against peptic barrier disruption, yet the molecular mechanisms of injury and protection were unclear. Herein, Barrett’s esophageal (BAR-T) cells were pretreated with buffered saline (HBSS; control), dilute alginate medications (Gaviscon Advance or Gaviscon Double Action, Reckitt Benckiser), a viscosity-matched placebo, or ADAM10 and matrix metalloproteinase (MMP) inhibitors before exposure to HBSS pH7.4 or pH4 ± 1 mg/mL pepsin for 10–60 min. Cell viability was assessed by ATP assay; mediators of epithelial integrity, E-cadherin, ADAM10, and MMPs were examined by Western blot and qPCR. Alginate rescued peptic reduction of cell viability (*p* < 0.0001). Pepsin-pH4 yielded E-cadherin fragments indicative of regulated intramembrane proteolysis (RIP) which was not rescued by inhibitors of known E-cadherin sheddases. Transcriptional targets of E-cadherin RIP fragments were elevated at 24 h (*MMP-1,2,9,14*; *p* < 0.01). Alginate rescued E-cadherin cleavage, ADAM10 maturation, and MMP induction (*p* < 0.01). Results support RIP as a novel mechanism of peptic injury during GERD. Alginate residue after wash-out to mimic physiologic esophageal clearance conferred lasting protection against pepsin-induced molecular mechanisms that may exacerbate GERD severity and promote carcinogenesis in the context of weakly acidic reflux.

## 1. Introduction

Gastroesophageal reflux disease (GERD), characterized by its hallmark symptoms, heartburn and/or regurgitation, is one of the most common gastrointestinal disorders managed by gastroenterologists and primary care physicians. The incidence of GERD is high and increasing, with rates of 13–20% in several high-income countries [1,2,3,4]. Its prevalence, impact on quality of life, and typical requirement for long-term treatment consume substantial economic and health care resources. The 2015 direct economic impact of GERD and its complications in the US alone was estimated at >$18 billion, 70% of which was attributed to proton pump inhibitor (PPI) therapy, and substantial indirect costs are attributed to reduced productivity and time off work [5,6]. GERD increases risk of metaplasia (Barrett esophagus; BE) which is histologically confirmed in 3–14% of GERD patients globally, although the true prevalence is likely much higher [7,8]. Associated esophageal adenocarcinoma (EAC) has increased rapidly in recent decades and <20% survive 5 years [9,10].

Acid-suppressing PPIs have been the first-line therapy in GERD for over three decades and represent one of the most commonly prescribed drug classes in Western countries, in use by up to 35% of adults and >50% of older age groups [11,12,13,14,15]. Potentially inappropriate off-label indications represent more than half of all prescriptions and the availability of low-cost, over-the-counter PPIs and patient perception of the drugs as safe contribute to additional nonprescription use [16]. While widely used, PPIs provide insufficient symptom relief in 20–40% of patients with esophagitis and perform even poorer in patients with non-erosive reflux disease [17,18,19]. Their chemopreventive benefit is controversial [20], with several large retrospective case-control studies finding PPI use, particularly long-term high adherence, positively correlated with esophageal adenocarcinoma [21,22,23]. PPI therapy reduces the acidity, but not frequency, of refluxate [24] and may increase the concentration or toxicity of other refluxate constituents, pepsin and bile [25,26,27]. Clinicians and patients are increasingly seeking alternatives due to burgeoning evidence of their associated risks [28].

Alginate has a long history of use as a monotherapy for mild-to-moderate GERD and a complimentary therapy for PPI-refractory GERD [29,30]. Alginate improves endoscopic findings [29,31,32,33] and relieves moderate-to-severe GERD symptoms with an efficacy similar to PPIs [30] yet a superior safety profile given its non-systemic activity. Alginate-antacid antireflux medications display immediate action, forming a raft which floats over stomach contents, eliminating or displacing the postprandial “acid pocket” such that the raft is preferentially refluxed, thereby reducing acidic reflux events [34,35,36]. Alginate inactivates pepsin and sequesters pepsin and bile salts, preventing esophageal contact [34,37]. Alginate is also an excellent mucoadhesive, recently found to preserve epithelial barrier integrity and cellular adhesion during prolonged acid–pepsin challenge in vitro [38,39,40]. Absent alginate pretreatment, peptic barrier disruption occurring within an hour in these experiments was speculated to arise by cytotoxicity or damage to adhesion molecules [41,42,43,44,45,46]. Cleavage of adherens junction protein E-cadherin has been observed in GERD biopsies [47] and E-cadherin proteolysis promotes over-expression of matrix metalloproteinases (MMPs), which further disrupt epithelial integrity and contribute to GERD severity, Barrett’s esophagus and esophageal cancer [48,49,50,51,52,53]. The aim of this study was to investigate the effects of acidified pepsin on molecular mediators of epithelial integrity, E-cadherin, and MMPs, and protection against these changes by alginates.

## 2. Results

### 2.1. Cell Viability

**Figure 1:**
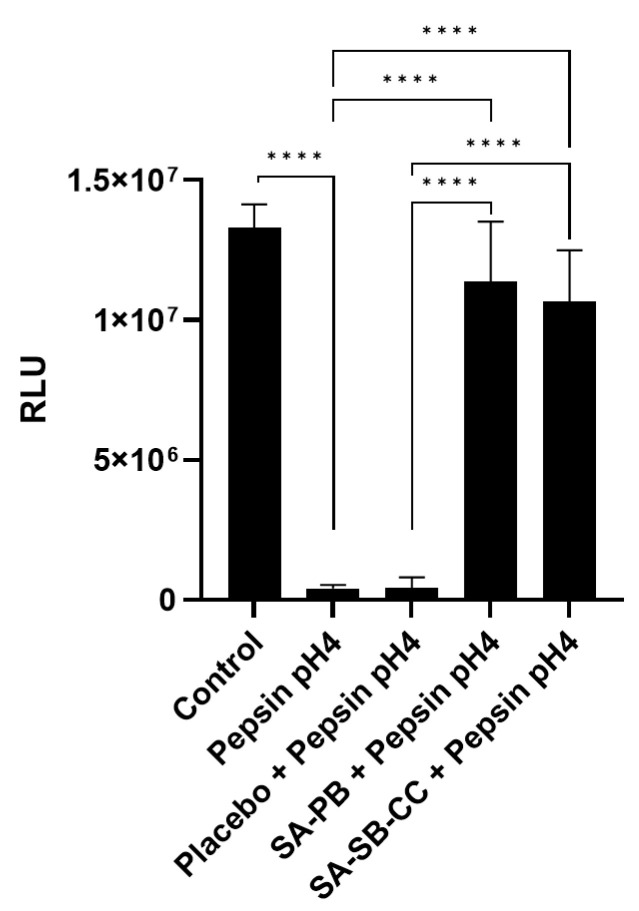
Effect of 1 h pepsin-acid treatment on cell viability and protection by alginate. Statistical analysis was performed on 8 replicates (*n* = 8) per condition, presented as mean ± SD, using multiple Student’s *t*-tests for comparisons between two groups. Conditions (pretreatment/treatment): Control (HBSS/HBSS); Pepsin pH4 (HBSS/pepsin pH4); Placebo + Pepsin pH4 (placebo/pepsin pH4), SA-PB + Pepsin pH4 (sodium alginate potassium bicarbonate/pepsin pH4), SA-SB-CC + Pepsin pH4 (sodium alginate, sodium bicarbonate, calcium carbonate/pepsin pH4). RLU = Relative luminescent units; error bars = SD; **** *p* < 0.0001.

Brief 1 min pretreatment with an undilute placebo or alginate caused the loss of cell viability, presumably due to viscosity occluding cell respiration. A 1:10 dilution, more representative of physiologic conditions wherein swallowing promotes esophageal clearance [38,54], attenuated the loss of cell viability and was, therefore, used throughout (Supplemental Figure S1). Under these conditions, pepsin in weak acid (pH4) drastically reduced cell viability at 1 h which was rescued by alginate (*p* < 0.0001) but not a viscosity-matched placebo (Figure 1). No significant difference was observed between the relative rescue effects of alginate formulations. A 10–30 min pepsin pH4 treatment produced minimal loss of viability (Supplemental Figure S2) and, hence, was used for follow-up analyses of molecular mediators of epithelial integrity.

### 2.2. E-Cadherin and ADAM-10 Cleavage

**Figure 2:**
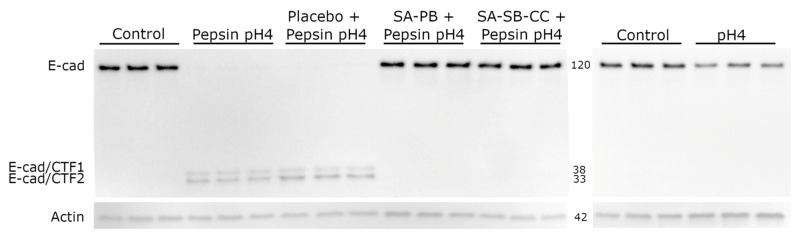
Effect of 30 min pepsin-acid treatment on E-cadherin regulated intramembrane proteolysis (RIP) and protection by alginate. Conditions (pretreatment/treatment): Control (HBSS/HBSS); Pepsin pH4 (HBSS/pepsin pH4); Placebo + Pepsin pH4 (placebo/pepsin pH4), SA-PB + Pepsin pH4 (sodium alginate potassium bicarbonate/pepsin pH4), SA-SB-CC + Pepsin pH4 (sodium alginate, sodium bicarbonate, calcium carbonate/pepsin pH4), pH4 (HBSS/HBSS pH4). E-cad = E-cadherin, CTF = C-terminal fragment.

**Figure 3:**
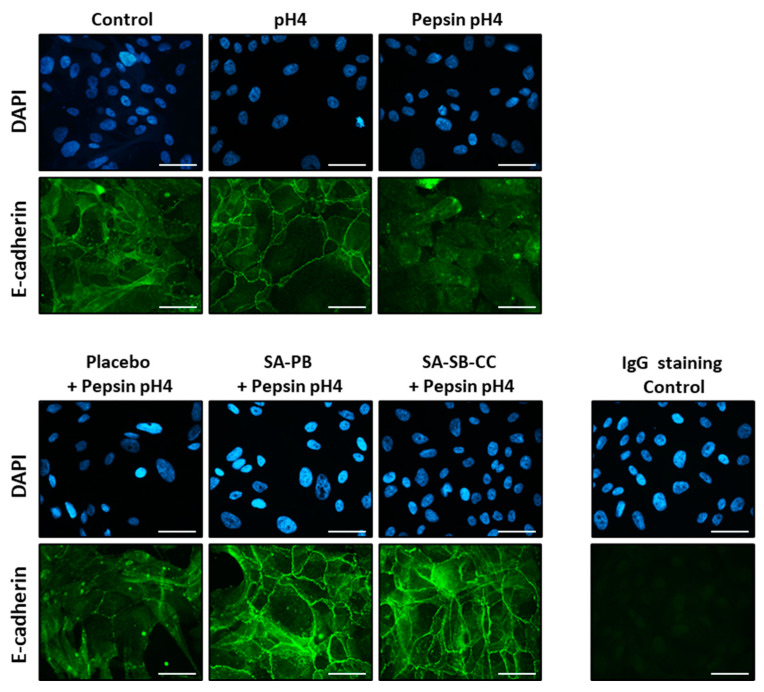
Effect of 30 min pepsin-acid treatment on membrane-localized E-cadherin and protection by alginate. Conditions (pretreatment/treatment): Control (HBSS/HBSS); pH4 (HBSS/HBSS pH4); Pepsin pH4 (HBSS/pepsin pH4); Placebo + Pepsin pH4 (placebo/pepsin pH4), SA-PB + Pepsin pH4 (sodium alginate potassium bicarbonate/pepsin pH4), SA-SB-CC + Pepsin pH4 (sodium alginate, sodium bicarbonate, calcium carbonate/pepsin pH4). Scale bar = 50 μm.

Pepsin-acid (1 mg/mL pepsin, pH4) treatment yielded E-cadherin fragments consistent with regulated intramembrane proteolysis (RIP) within 10 min (Supplemental Figure S3). Pepsin-acid cleaved nearly all full-length E-cadherin (120 kDa) by 30 min (Figure 2), resulting in 38 and 33 kDa C-terminal E-cad/CTF1 and E-cad/CTF2 fragments indicative of RIP. Acid alone did not induce E-cadherin cleavage. Alginate (but not a viscosity-matched placebo) rescued pepsin-acid-mediated E-cadherin cleavage. Immunofluorescent staining demonstrated depletion of membrane-associated E-cadherin ectodomain by 30 min pepsin-acid treatment (Figure 3). E-cadherin cleavage and translocation were rescued by alginate but not the placebo and were not caused by acid alone (Figure 2 and Figure 3).

**Figure 4:**
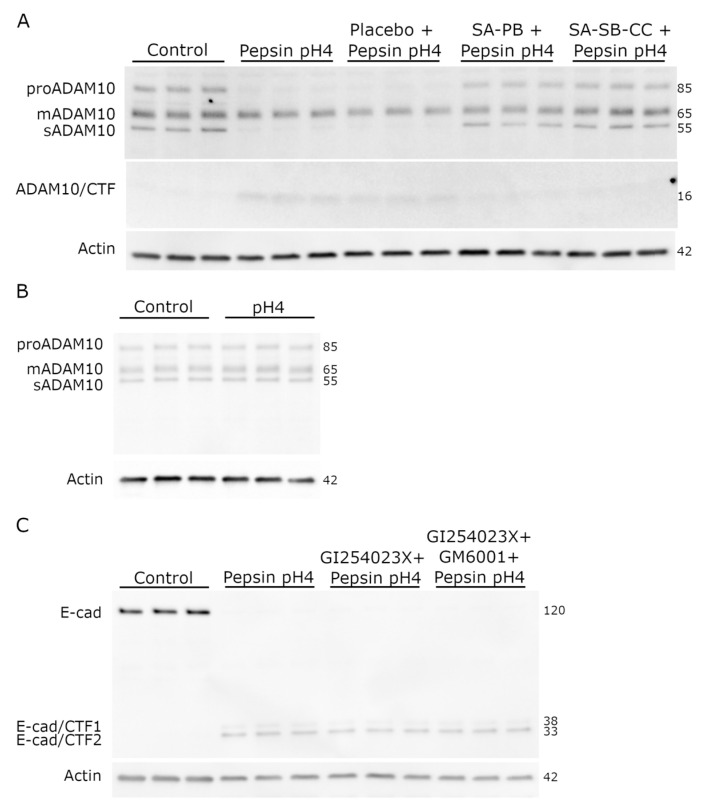
Role of known E-cadherin sheddases, ADAM10 and metalloproteinases, in pepsin-induced E-cadherin RIP. (**A**,**B**) Effects of 30 min pepsin-acid treatment and alginate pretreatment on maturation of ADAM10, a major E-cadherin sheddase. Conditions (pretreatment/treatment): Control (HBSS/HBSS); Pepsin pH4 (HBSS/pepsin pH4); Placebo + Pepsin pH4 (placebo/pepsin pH4), SA-PB + Pepsin pH4 (sodium alginate potassium bicarbonate/pepsin pH4), SA-SB-CC + Pepsin pH4 (sodium alginate, sodium bicarbonate, calcium carbonate/pepsin pH4), pH4 (HBSS/HBSS pH4). (**C**) Effect of inhibition of ADAM10 (by GI254023X) and other metalloproteinases (by GM6001, broad spectrum inhibitor) on pepsin-induced E-cadherin RIP. Conditions (pretreatment/treatment): Control (HBSS/HBSS); Pepsin pH4 (HBSS/pepsin pH4); GI254023X + Pepsin pH4 (GI254023X/pepsin pH4 + GI254023X); GI254023X + GM6001 + Pepsin pH4 (GI254023X + GM6001/pepsin pH4 + GI254023X + GM6001). mADAM10 = mature ADAM10, sADAM10 = ADAM10 secreted ectodomain, E-cad = E-cadherin, CTF = C-terminal fragment.

Pepsin-acid treatment (1 mg/mL pepsin pH4, 30 min), but not acid alone, caused ADAM10 maturation (depleted proADAM10 and relative abundance of mADAM10), which was rescued by alginate but not the placebo (Figure 4). The RIP cleavage fragment of ADAM10, secreted ectodomain (sADAM10), was depleted by pepsin and rescued by alginate, while 16 kDa ADAM10/CTF (intermediate of ADAM10 RIP) accumulated suggesting inhibition of its γ-secretase cleavage; these effects were also rescued by alginate but not the placebo. Pretreatment with ADAM10 inhibitor, alone or combined with MMP inhibitor, did not prevent E-cadherin RIP.

### 2.3. MMP Expression

**Figure 5:**
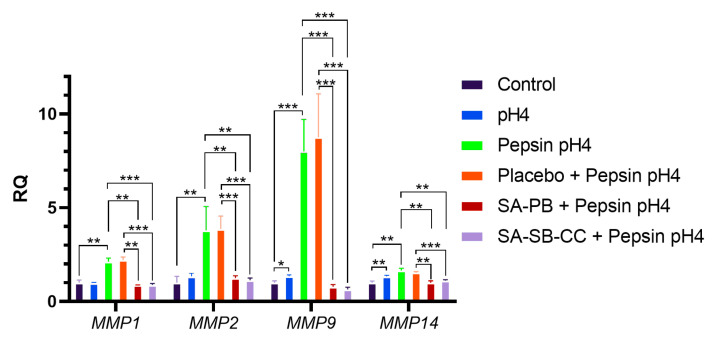
Effect of 15 min pepsin-acid stimulation on MMP mRNA expression 24 h post-treatment and protection by alginate. Statistical analysis was performed on 3 replicates (*n* = 3) per condition, presented as mean ± SD, using multiple Student’s t-tests for comparisons between two groups. Conditions (pretreatment/treatment): Control (HBSS/HBSS); pH4 (HBSS/HBSS pH4); Pepsin pH4 (HBSS/pepsin pH4); Placebo + Pepsin pH4 (placebo/pepsin pH4), SA-PB + Pepsin pH4 (sodium alginate potassium bicarbonate/pepsin pH4), SA-SB-CC + Pepsin pH4 (sodium alginate, sodium bicarbonate, calcium carbonate/pepsin pH4). RQ = Relative quantity; error bars = SD; *** *p* < 0.001; ** *p* < 0.01; * *p* < 0.05.

The expression of MMPs transcriptionally regulated by E-cadherin cleavage fragments (*MMP-2,9*, and *14*) [52] or harboring association with reflux-attributed cancer (*MMP1*) were upregulated by acidified pepsin 24 h following 15 min stimulation; induction was rescued by alginate but not the placebo (Figure 5 and Supplemental Table S1). The two alginate formulations exhibited similar protection. Baseline expressions of MMPs were similar: the mean relative quantity ± SD *MMP1* = 1.21 ± 0.18; *MMP2* = 1.35 ± 0.46; *MMP9* = 0.98 ± 0.10; and *MMP14* = 0.92 ± 0.08.

### 2.4. Proliferation Assay

**Figure 6:**
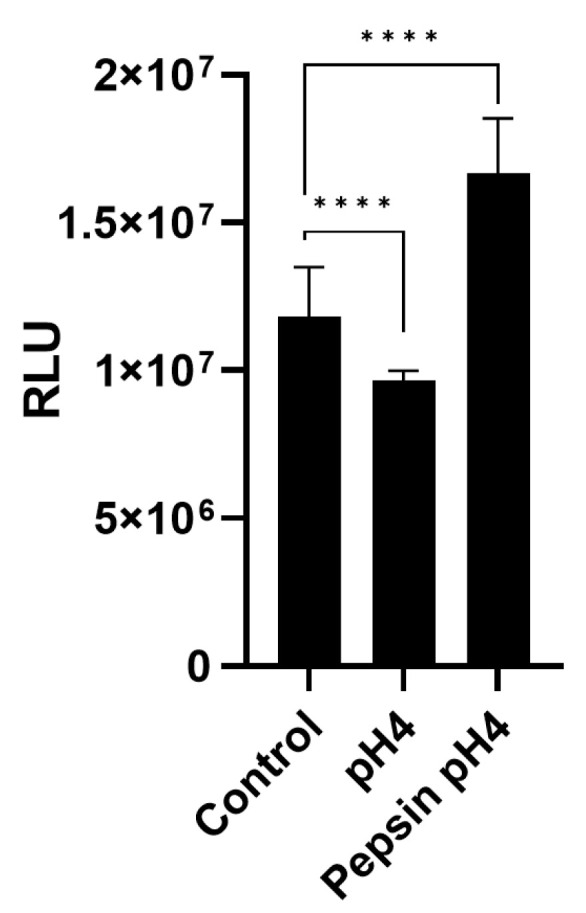
Effect of 15 min pepsin-acid stimulation on cell proliferation over 48 h. Statistical analysis was performed on 24 replicates (*n* = 24) per condition, presented as mean ± SD, using multiple Student’s t-tests for comparisons between two groups. RLU = Relative luminescent units; error bars = SD; **** *p* < 0.0001.

ATP bioluminescence was used to compare number of viable cells across treatment groups seeded at equal densities and allowed to proliferate for equal duration as an indicator of cell proliferation [55]. Fifteen-minute pepsin-acid treatment caused a significant increase, and acid caused a significant decrease, in esophageal cell proliferation as indicated by cellular ATP measured in wells seeded equally 3 h post-treatment and cultured under normal growth conditions for 48 h (*p* < 0.0001; Figure 6).

## 3. Discussion

Local treatment or prevention of esophageal injury due to GERD has long been an area of interest to which the brief transit time of orally administered drugs (<16 s) has represented a major challenge [56]. Early comparisons of widely utilized pharmaceutical mucoadhesives found sodium alginate provided superior esophageal adhesion [57], and years of its diverse applications since have led to a growing appreciation of its topical protective capacities: impedance of chemical diffusion preventing epithelial contact and cytotoxicity, promotion of wound healing, sequestration of inflammatory mediators (cytokines, reactive oxygen species), and neutralization of molecular and chemical aggressors including pepsin, acid, and bile [34,58,59,60,61]. Peptic inhibition by alginates is dose-dependent and non-competitive (cannot be overcome by high concentrations of substrate) [62]. The efficacy of peptic inhibition by isolated alginate polymers, including that of Gaviscon products, has been characterized and demonstrates correlation with structure; an alternating mannuronic and guluronic acid composition provides superior inhibition, possibly due to improved acid solubility or binding to the active site of pepsin [62].

Alginate-based anti-reflux medications confer GERD symptom relief for up to 4 h, the duration of raft stability in the stomach [29,30,63]. While >75% of the mucosal impedance conferred by alginate is lost 10 min post-consumption, a thin film of mucoadhesive alginate is thought to persist and the esophagus may be continually bathed in alginate as the raft overlaying gastric contents is preferentially refluxed [54]. Such a thin layer of residual alginate has been shown to provide up to 90 min topical protection in vitro and ex vivo under conditions that mimic swallowing; a viscosity-matched placebo demonstrated neither mucoadhesion nor protection against epithelial barrier dysfunction by refluxate constituents [38,40]. Herein, residue remaining after the wash-out of dilute alginate-based antireflux medications provided dramatic protection of esophageal cell viability against physiological concentrations of acidified pepsin for 60 min. The inefficacy of the placebo herein and previously [38,39,40] support protection by alginate rather than solution viscosity.

The acutely erosive nature of acidified pepsin has historically been attributed to digestion of protein constituents of the mucin layer, basement membrane, and intercellular junctions [45,64,65,66]. E-cadherin was chosen for study herein given its cleavage near the cell membrane in GERD biopsies [47]. E-cadherin is the primary constituent of adherens junctions which are critical to tissue integrity, immobilizing cells within the epithelium and facilitating contact inhibition of growth, and essential to the function of tight junctions, the primary permeability barrier of the epithelium [67,68]. In alignment with the evolving model of GERD pathogenesis from simple caustic injury towards a complex array of refluxate-mediated molecular signaling events, [41] we found that rather than merely degrading E-cadherin, acidified pepsin initiated its RIP as indicated by the persistence of specific cleavage fragments. RIP is a relatively recently identified, yet evolutionarily conserved cell signaling strategy in which an integral membrane protein is cleaved near the cell surface by a sheddase, triggering subsequent cleavage by an intramembrane-cleaving protease and the liberation of dormant signaling molecules of defined biological functions [69]. E-cadherin RIP fragments harbor activities related to cell adhesion, apoptotic resistance and proliferation. The N-terminal ectodomain fragment (80 kDa soluble E-cadherin, sE-cad), diffuses into the extracellular environment where it may occlude pairing of intact adhesion-competent E-cadherin molecules; chemotactically anchor E-cadherin on migrating cells; promote proliferation and apoptotic resistance via growth factor receptor signaling pathways; and upregulate MMPs which further destabilize epithelial integrity [52,70,71]. The remaining integral membrane C-terminal fragment of E-cadherin (38 kDa E-cad/CTF1) is then cleaved by a γ-secretase (presenilin-1/2), releasing 33-kDa E-cad/CTF2 to the cytosol. [70] This liberates β-catenin which facilitates oncogenic Wnt signaling, whose transcriptional targets include E-cadherin sheddase, MMP-7 [72], and p120 catenin, which promotes E-cad/CTF2 DNA-binding and apoptotic resistance via Kaiso [72]. E-cadherin RIP (serum s-E-cad) is associated with various cancers [70] and E-cadherin depletion is correlated with pepsin presence in the larynx of LPR patients and observed in GERD/laryngopharyngeal reflux-attributed metaplasia and cancer and other aerodigestive conditions associated with impaired barrier integrity [73,74,75,76,77].

E-cadherin RIP fragments (~35 kDa CTF and sE-cad) have been observed in GERD patient esophageal biopsies and serum, respectively, yet absent (CTF) or reduced (sE-cad) in healthy control subjects [47]. ADAM10, a major E-cadherin sheddase [78], was implicated in E-cadherin RIP by its colocalization and maturation in GERD biopsies and was speculated to have been activated by refluxed acid. While some MMPs are acid-activated [53], maturation and activation of ADAM10 is incompletely understood, requiring membrane perturbation (as triggered by Ca+ influx or apoptotic stimuli) and the reorganization of the enzyme cofactor, phosphatidylserine [79]. ADAM10 activity is further modulated via RIP [80,81,82,83]. Where acid alone has been shown to elicit E-cadherin cleavage in aerodigestive tract epithelia, the effect is delayed, observed after 24 h, and secondary to transcriptional regulation of MMPs [84,85]. MMP-7 specifically was implicated in acid-induced E-cadherin RIP during LPR: pharyngeal E-cadherin depletion and MMP-7 accumulation were associated with positive reflux symptom index in LPR patients and in vitro experiments demonstrated that weak acid (pH4, 5 min) induced E-cadherin RIP in pharyngeal cells 24 h post-exposure dependent upon de novo synthesis of MMP-7 [85,86]. However, treatment with acid alone is not representative of GERD or LPR, as all refluxate contains pepsin and the physiological relevance is questionable given that acid > pH2 does not reduce barrier integrity in vivo or ex vivo but requires pepsin or bile salts [27,43,44,45,87]. To our awareness, the immediate effects of acidified pepsin on E-cadherin RIP in aerodigestive tract epithelia and involved sheddases have not been examined to date.

Herein, acidified pepsin elicited E-cadherin RIP within 10 min, suggesting that pepsin activates an already-present sheddase or functions as the sheddase itself. Acid alone did not alter relative abundance of mADAM10 or induce E-cadherin RIP despite the presence of mADAM10. While ADAM10 is considered the predominant E-cadherin sheddase [78], a diverse array of endogenous and exogenous cysteine, serine, and aspartic proteases serve as E-cadherin sheddases as well, including integral membrane (MMP-3,7,9,14,15 and ADAM-10,12,15), blood (plasmin, kallikrein), and bacterial proteases (*HtrA* expressed by *Helicobacter pylori*) [70,88]. Although acidified pepsin led to a relative abundance of mADAM10 herein, E-cadherin RIP was not prevented by the specific ADAM10 inhibitor GI254023X alone or in combination with the broad-spectrum metalloproteinase inhibitor GM6001, which together would inhibit the majority if not all currently recognized epithelial sheddases of E-cadherin. E-cadherin RIP was, however, rescued by alginate-based anti-reflux medications which inhibit pepsin. While it cannot be definitively concluded that pepsin was the E-cadherin sheddase based on these experiments, the data strongly support the possibility. As sheddase or sheddase activator, pepsin-induced RIP during GERD likely extends beyond E-cadherin. Additional GERD/EAC-relevant molecules that undergo RIP include LRP1 (putative pepsin receptor [89]), TNFA, OPA1 (whose RIP results in apoptotic resistance and mitochondrial cristae remodeling [90] similar to that induced by pepsin [91,92,93]), CD44 (adhesion molecule elevated in esophageal cancer [94]), EpCAM (adhesion molecule and prognostic indicator of esophageal cancer [95]), and Notch (role in EAC [96]). This intriguing possibility warrants further research. The capacity of alginate to protect against pepsin-induced E-cadherin RIP demonstrated herein may be clinically important given that E-cadherin has been proposed as a biomarker for GERD and its loss alone is sufficient to increase junctional permeability [47].

The mechanisms of peptic injury during GERD and LPR demonstrate significant overlap with the known biological functions of E-cadherin RIP fragments: hyperproliferation, dysregulated cell migration, altered wound healing, EGFR activation, change in cell phenotype, and apoptotic resistance [65,74,75,91,92,93,97,98,99]. The data herein corroborate previously demonstrated antiproliferative effects of acid pH4 alone [100,101] and reports of pepsin-induced epithelial hyperproliferation and epithelial-mesenchymal transition which is characterized by elevated MMP expression [74,75,89,91,92,97,102,103]. The consequences of MMP dysregulation include impaired epithelial integrity and promotion of immune cell invasion, cell migration, and metastatic potential [53]. The pattern of MMP induction by pepsin herein was consistent with that elicited by sE-cad in lung cells: [52] *MMP-2,9,14* were elevated with the greatest increase in *MMP9*. MMP-9 is elevated in mild and severe GERD and plays a role in the early inflammatory response in a surgical BE model [48,49]. Among MMPs, MMP-14 is considered to harbor the greatest influence over the critical balance between cell adhesion and extracellular matrix (ECM) proteolysis: in addition to cleavage of basement membrane and ECM proteins, it cleaves important cell-cell and cell-matrix adhesion molecules such as integrin, activates other MMPs, is involved in release of signaling molecules including transforming growth factor β and cytokines, and demonstrates strong association with the invasion, migration, and angiogenesis of aerodigestive tract cancers [104]. Pepsin also induced *MMP1*, a pre-invasive factor for BE associated with GERD severity and EAC [50,51]. Importantly, alginate pretreatment prevented MMP induction by pepsin.

This study demonstrates that alginate-based antireflux medications inhibit E-cadherin proteolysis, a phenomenon observed in GERD and LPR biopsies which is sufficient to cause barrier dysfunction. Experiments herein demonstrate that E-cadherin RIP was caused by acidified pepsin but not acid alone, and that antireflux medications containing a known pepsin inhibitor, alginate, prevent E-cadherin RIP, whereas inhibitors of other E-cadherin sheddases did not. Alginate-based medications further prevented pepsin-acid-induced MMP expression, which is triggered by E-cadherin RIP and exacerbates loss of epithelial integrity. The contribution of a number of MMPs to GERD-attributed EAC risk suggests a comprehensive survey of pepsin-responsive MMPs in esophageal epithelia is warranted [50]. In addition, while the increase in cell number 48 h post-treatment caused by pepsin-acid herein corroborates pepsin-induced hyperproliferation as reported in the prior literature, more informative nucleoside analog incorporation and flow cytometric cell cycle analysis will be used in future experiments investigating alginate rescue of pepsin-acid-mediated effects on cell cycle progression. Additional experiments examining potential pepsin-acid-induced cytotoxicity, cell migration, wound healing, and apoptosis or apoptotic resistance and rescue by alginate are also warranted. Further experiments using isolated sE-cad, [52] antibodies blocking E-cadherin cleavage [78], and pharmacologic inhibitors of β-catenin/Wnt signaling would provide additional insight regarding the contributions of sE-cad and E-cad/CTF2 to MMP regulation and hyperproliferation by acidified pepsin. Finally, alginate has exhibited efficacy for LPR symptoms and prevents pepsin-mediated disruption of laryngeal epithelial integrity [40,105], which we recently found similarly involves E-cadherin RIP (data submitted for publication [106]); hence, alginate rescue of laryngeal E-cadherin RIP will be examined as well.

Limitations of this study include its basis on a single-cell culture model. While cell lines are invaluable to scientific investigation, caution should be exercised when extrapolating findings to clinical situations. Within the constraints of the model, best attempts were made to mimic the physiological conditions of GERD. Human gastric fluid is pH1.5–3 containing ~0.5–1 mg/mL pepsin [107,108] which may be concentrated more than an order of magnitude given PPI-suppressed acid secretion [25,27]. In untreated GERD, refluxate is predominantly acidic (63% pH < 4), giving rise to >80% of symptom episodes [109]. Provided PPIs, refluxate is commonly (80%) pH ≥ 4, giving rise to 72% of symptom episodes [110]. A wash-out of dilute alginate mimicked the physiologic conditions of esophageal clearance [38,54]. This study provides useful information that may inform the design of future in vivo and clinical studies. Follow-up experiments are planned using esophageal biopsy specimens collected from patients with varying GERD severity to address differences in sensitivity to pepsin-acid and protection by alginates across GERD phenotypes.

In summary, the data herein add to an expanding body of work implicating pepsin in reflux-attributed inflammation and carcinogenesis of the aerodigestive tract [74,75,91,92,93,97,98,99]. The protective benefits of alginate against E-cadherin RIP and MMP dysregulation, which are in turn associated with GERD severity and carcinogenesis, are particularly intriguing given that the current mainstay treatment for severe GERD, PPIs, have failed to demonstrate chemopreventive benefit or stem the rising incidence of EAC despite their widespread use. Whereas PPI therapy may increase the concentration or toxicity of refluxate constituents such as pepsin and bile [25,26,27], alginate-based antireflux medications neutralize acid and inactivate and sequester pepsin and bile, providing protection throughout the average duration of postprandial reflux [34,35,36,111]. While PPIs provide valuable relief for GERD symptoms and esophagitis, the data herein suggest that alginate adjunctive therapy may prevent exacerbation of GERD severity and provide chemoprotective benefit for patients at risk of BE and EAC. Future work is warranted to examine these possibilities.

## 4. Materials and Methods

### 4.1. Cell Culture

hTERT-immortalized Barrett’s esophageal cells BAR-T [112] (the kind gift of Rhonda Souza) were cultured as they were previously [40]. Briefly, cells were cultured in modified Keratinocyte Growth Media 2 (Lonza, Walkersville, MD, USA), including the provided hydrocortisone, insulin and transferrin supplements, with 180 μM adenine and 10 ng/mL cholera toxin (both Sigma-Aldrich, St. Louis, MO, USA) and 70 μg/mL bovine pituitary extract, 5% fetal bovine serum, and 1× Antibiotic-Antimycotic (each ThermoFisher Scientific, Waltham, MA, USA) on collagen-I coated flaskware (Biocoat; Corning, Corning, NY, USA). Cells were cultured to 90% confluence for examining protein expression and 50% for gene expression.

Generic designations include Gaviscon Advance^®^ (Reckitt Benckiser Group, Slough, UK; 1000 mg sodium alginate and 200 mg potassium hydrogen carbonate per 10 mL) as “SA-PB”, and Gaviscon Double Action^®^ (Reckitt Benckiser Group; 500 mg sodium alginate, 213 mg sodium bicarbonate, 325 mg calcium carbonate per 10 mL) as “SA-SB-CC”. The placebo consisted of xanthan gum solution of matching viscosity absent alginate or bicarbonates [38].

In triplicate unless noted, cultures were pretreated for 1 min in HBSS (pH7.4; control, ThermoFisher Scientific, Waltham, MA, USA) or 1:10 dilutions of SA-PB, SA-SB-CC, or a placebo in HBSS. Cultures were washed twice in HBSS and treated in HBSS pH7.4 or HBSS pH4 ± 1 mg/mL porcine pepsin (Sigma-Aldrich) at 37 °C/5% CO_2_ for 15 (qPCR), 30 (immunofluorescence or Western blot), or 60 min (ATP cell viability assay).

### 4.2. ATP Assay

To test viability, cultures at 75% confluence were treated as above in 8 replicates. Specifically, cells were pretreated (*n* = 8) for 1 min in HBSS (pH7.4 control) or 1:10 dilutions of placebo or alginate-based antireflux medications. Cultures were washed twice in HBSS and treated in HBSS pH7.4 or HBSS pH4 ± 1 mg/mL porcine pepsin at 37 °C/5% CO_2_ for 60 min. ATP (indicator of viable cells) was assessed immediately post-treatment per manufacturer instructions (CellTiter-Glo^®^ 2.0, Promega, Madison, WI, USA), specifically, lysed in 1:1 assay reagent in HBSS for 10 min before a luminescence measurement using a Tecan Spark microplate reader (Tecan, Männedorf, Switzerland).

ATP bioluminescence was also used as an indicator of cell proliferation [55]. Cells were treated in triplicate with HBSS or HBSS pH4 ± 1 mg/mL pepsin at 37 °C/5% CO_2_ for 15 min, washed twice in HBSS, and incubated in growth media at 37 °C/5% CO_2_ for 3 h, then trypsinized and 1E4 cells distributed in 8 replicates per treatment triplicate in a 96-well plate cultured at 37 °C/5% CO_2_ for 48 h prior to assay.

### 4.3. Western Blot

To examine E-cadherin cleavage, cells were pretreated in triplicate wells for 1 min in HBSS (pH7.4 control) or 1:10 dilutions of placebo or alginate-based antireflux medications. Cultures were washed twice in HBSS and treated in HBSS pH7.4 or HBSS pH4 ± 1 mg/mL porcine pepsin at 37 °C/5% CO_2_ for 30 min and immediately harvested and lysed.

To examine the acute effects of pepsin-acid and alginate on maturation of A Disintegrin and metalloproteinase domain-containing protein 10 (ADAM10), a major E-cadherin sheddase, cells were pretreated (*n* = 3) for 1 min in HBSS (pH7.4 control) or 1:10 dilutions of placebo or alginate-based antireflux medications. Cultures were washed twice in HBSS, treated in HBSS pH7.4, or HBSS pH4 ± 1 mg/mL porcine pepsin at 37 °C/5% CO_2_ for 30 min and immediately harvested and lysed.

To test MMPs as E-cadherin sheddases, cells were pretreated for 2 h with 20 uM GI254023X (Sigma-Aldrich; specific inhibitor of ADAM10) ± 20 uM GM6001 (Sigma-Aldrich; broad-spectrum metalloproteinase inhibitor) or DMSO (solvent) in HBSS. Half-maximal inhibitory concentration values reported for GM6001 against human MMPs are: MMP-1 (6 nmol/L), MMP-2 (7/17 nmol/L), MMP-3 (28 nmol/L), MMP-7 (41 nmol/L), MMP-8 (1.4 nmol/L), MMP-9 (4.1/15 nmol/L), MMP-12 (23 nmol/L), MMP-13 (3.2 mmol/L), MMP-14 (23/33 nmol/L), MMP-15 (6 nmol/L), MMP-16 (8 nmol/L), and MMP-26 (17 nmol/L; and inhibition of MMP-10, MMP-17, MMP-20, MMP-21, TACE, ADAM19, other ADAMs, anthrax lethal factor, neprilysin, leucine aminopeptidase, and DPPIII has also been reported [113,114,115]. GI254023X exhibits selective activity for ADAM10; it has >100-fold higher potency towards ADAM10 compared to ADAM17. Cells were then treated in HBSS with DMSO or 1 mg/mL pepsin in HBSS pH4 with DMSO, 20 uM GI254023X, or 20 uM GI254023X + 20 uM GM6001 at 37 °C/5% CO_2_ for 30 min.

Treatment solutions were collected and 2 uM NaOH and Halt™ Protease Inhibitor Cocktails (ThermoFisher Scientific) were added. Cells were harvested in cold lysis buffer (1% NP40, 1 mM EDTA, 150 mM NaCl, 20 mM Tris-Cl, pH7.4) containing protease inhibitor. To examine ADAM10 expression, the lysis buffer contained 10 uM GI254023X to prevent maturation upon lysis [116]. Proteins were separated (4–20% TGX, Bio Rad Laboratories, Hercules, CA, USA) and transferred to polyvinylidene difluoride. Membranes were blocked (5% milk, 0.1% Tween-20, phosphate-buffered saline, PBS) and probed with antibodies to E-cadherin C-terminus (4A2C7; ThermoFisher Scientific), ADAM10 (AB19026, Sigma-Aldrich), or actin (CP01, Sigma-Aldrich) for 1.5 h at room temperature. Membranes were washed in three changes of PBS + 0.1% Tween-20 for 45 min and incubated in HRP-conjugated, affinity-isolated, polyclonal goat anti-mouse (P0447; Agilent Technologies, Santa Clara, CA, USA) for 1 h at room temperature, prior to a 10 min wash in PBS + 0.1% Tween-20. SuperSignal™ West Femto Maximum Sensitivity Substrate (ThermoFisher Scientific) was applied and the signal captured using Azure Biosystems c600 Western blot imaging system (Azure Biosystems, Dublin, CA, USA).

### 4.4. Immunofluoresence

Cells were immunostained using an anti-E-cadherin N-terminal antibody and methods adapted from those described previously [117]. Briefly, cells were grown on collagen-coated coverslips (Neuvitro Corporation, Camas, WA). Cells were pretreated (in dilute SA-PB, SA-SB-CC or placebo in HBSS), washed, and treated (in HBSS pH7.4 or HBSS pH4 ± 1 mg/mL porcine pepsin) at 37 °C/5% CO_2_ for 30 min as described above. Cells were then fixed in 4% paraformaldehyde (Electron Microscopy Services, Hattfield, PA) in PBS for 15 min, washed twice in PBS, and blocked for 2 h in blocking buffer (PBS, 5% normal goat serum (Cell Signaling Technology, Danvers, MA, USA), 50-mM ammonium chloride), and then incubated in an anti-E-cadherin N-terminal antibody (HECD1; ThermoFisher Scientific) or mouse immunoglobulin (negative control; Santa Cruz Biotechnology, Dallas, TX, USA) in 1× PBS, 1% bovine serum albumin for 1.5 h at room temperature. Coverslips were washed three times in PBS and blocked for 30 min in blocking buffer with 0.1% Tween-20. Primary antibodies were detected by 1 h incubation with Alexa Fluor 488 goat anti-mouse IgG H+L (ThermoFisher Scientific) at room temperature. Slides were washed in PBS before a water rinse and mounting to slides using Prolong Diamond Antifade Reagent with DAPI (ThermoFisher Scientific). The experiment was performed in duplicate; representative images were collected at 630x magnification with constant exposure for biomarkers of interest using an Axioscope 5 microscope (ZEISS, Oberkochen, Germany).

### 4.5. Real Time qPCR

For analysis by qPCR, cells were pretreated (in dilute SA-PB, SA-SB-CC or placebo in HBSS), washed, and treated (in HBSS pH7.4 or HBSS pH4 ± 1 mg/mL porcine pepsin) at 37 °C/5% CO_2_ for 15 min as described above, then washed twice in HBSS and incubated in normal growth media for 24 h prior to harvest in TRIZOL (ThermoFisher Scientific). RNA was extracted and the purity and concentration assessed by UV spectroscopy. RNA was reverse transcribed using Superscript IV VILO (ThermoFisher Scientific). qPCR was performed in quadruplicate reactions in a Viia7 instrument per the manufacturer’s instructions using intron-spanning commercial primer/probe sets (Taqman gene expression assays, ThermoFisher Scientific): *HPRT1* Hs99999909_m1; *MMP1* Hs00899658_m1; *MMP2* Hs01548727_m1; *MMP9* Hs00957562_m1; *MMP14* Hs00237119_m1. Threshold cycle (Ct) values < 36 were used for analysis, and gene expression was normalized to housekeeping gene *HPRT1* which demonstrates < 1.5-fold change in normal versus malignant esophagus [118].

### 4.6. Statistical Analysis

A Student’s *t*-test was used to compare groups. For the ATP assay, ROUT (GraphPad Prism 9.5.1, Dotmatics, Boston, MA, USA) robust regression and outlier removal at conservative Q = 0.5% was applied before the *t*-test. For qPCR, RQs were calculated via the delta-delta method and log transformation applied before the *t*-test. *p* < 0.05 was considered significant.

## 5. Conclusions

Rather than merely degrading cellular adhesion molecules and basement membrane proteins, pepsin in weak acid (pH4, characteristic of patients taking PPIs) triggered complex molecular signaling mechanisms associated with epithelial barrier dysfunction, GERD severity and carcinogenesis. Data herein suggest that refluxed pepsin functions as a sheddase of E-cadherin, inducing MMP expression and hyperproliferation in accord with known biological functions of E-cadherin RIP fragments. Pepsin also elicited maturation of ADAM10, a cancer-associated sheddase. Mucoadhesive alginate remaining after a wash-out of 10-fold dilute alginate (to mimic physiologic esophageal clearance) conferred lasting protection against E-cadherin RIP, ADAM10 maturation, and MMP induction. These data indicate that alginate may prevent the exacerbation of GERD severity in patients taking PPIs and provide chemopreventive benefit, which is particularly intriguing in light of the failure of current GERD first-line medical therapy in this regard.

## Data Availability

The data presented in this study are available herein and in the Supplementary Materials.

## Supplementary Materials

The supporting information can be downloaded at: https://www.mdpi.com/article/10.3390/ijms24097932/s1.
