# Supplementary material for: Topical Alginate Protection against Pepsin-Mediated Esophageal Damage: E-Cadherin Proteolysis and Matrix Metalloproteinase Induction
Source: Int J Mol Sci. 2023 Apr 27;24(9):7932. doi: 10.3390/ijms24097932 (PMC10178445; doi:10.3390/ijms24097932)

SUPPLEMENTAL INFORMATION

TABLES

Table S1. Expression of sE-cad responsive *MMPs*

	Mean RQ±SD	p rel Control	p rel Pepsin pH4	p rel Placebo + Pepsin pH4	p rel SA-pB + Pepsin pH4
MMP1					
Control	1.00±0.15				
pH4	0.98±0.04	0.90	0.0002		
Pepsin pH4	2.12±0.19	0.002			
Placebo + Pepsin pH4	2.21±0.17		0.57		
SA-PB + Pepsin pH4	0.87±0.01		0.0035	0.0022	
SA-SB-CC + Pepsin pH4	0.88±0.08		0.0003	0.0002	0.92
MMP2					
Control	1.00±0.34				
pH4	1.33±0.17	0.19	0.007		
Pepsin pH4	3.79±1.27	0.0072			
Placebo + Pepsin pH4	3.86±0.70		0.85		
SA-PB + Pepsin pH4	1.24±0.13		0.0053	0.0008	
SA-SB-CC + Pepsin pH4	1.14±0.11		0.0038	0.0005	0.37
MMP9					
Control	1.00±0.10				
pH4	1.34±0.08	0.013	0.0002		
Pepsin pH4	8.02±1.69	0.0001			
Placebo + Pepsin pH4	8.77±2.30		0.73		
SA-PB + Pepsin pH4	0.78±0.12		0.0001	0.0002	
SA-SB-CC + Pepsin pH4	0.64±0.12		0.0001	0.0002	0.25
MMP14					
Control	1.00±0.09				
pH4	1.33±0.07	0.0095	0.013		
Pepsin pH4	1.65±0.12	0.0018			
Placebo + Pepsin pH4	1.54±0.05		0.25		
SA-PB + Pepsin pH4	1.00±0.10		0.0026	0.0025	
SA-SB-CC + Pepsin pH4	1.11±0.05		0.0016	0.0008	0.17

RQ=relative quantity

Figure S1. Dilution of alginate (1:10) remedied loss of cell viability caused by undilute alginate. BAR-T cells were exposed to HBSS, placebo, or alginate or dilute placebo/alginate briefly before two washes in HBSS and 30 minute incubation in HBSS at 37°C/5% CO_2 prior to ATP cell viability assay. RLU=relative luminescent units; error bars=SEM

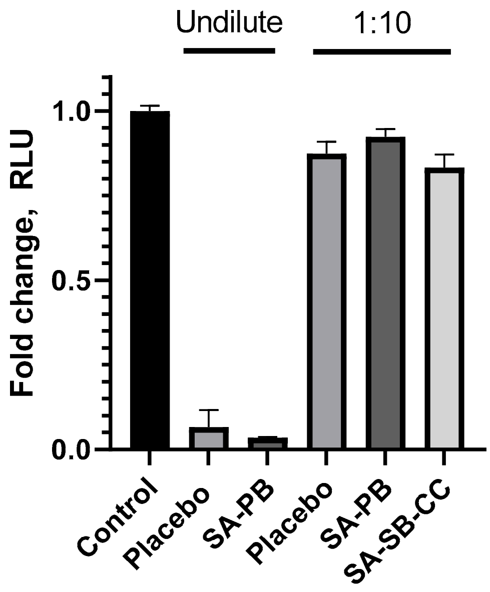

Figure S2. Pepsin-acid treatment (1mg/ml pepsin pH4) 10-30min yielded less drastic loss of cell viability relative to 1 hour treatment and was therefore used in experiments to assess gene/protein expression. RLU=relative luminescent units; error bars=SEM

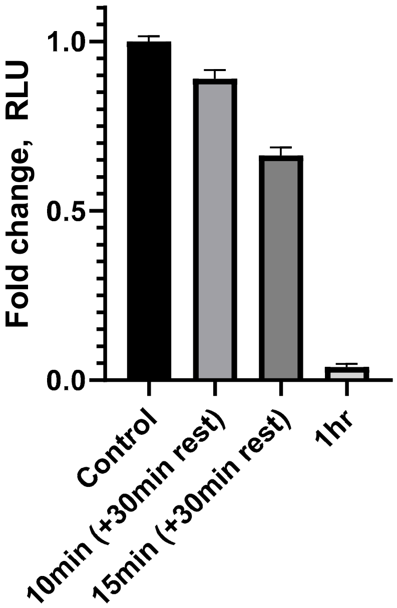

Figure S3. E-cadherin cleavage was partial after 10 minutes with 1mg/ml pepsin pH4 and total after 30 minutes.

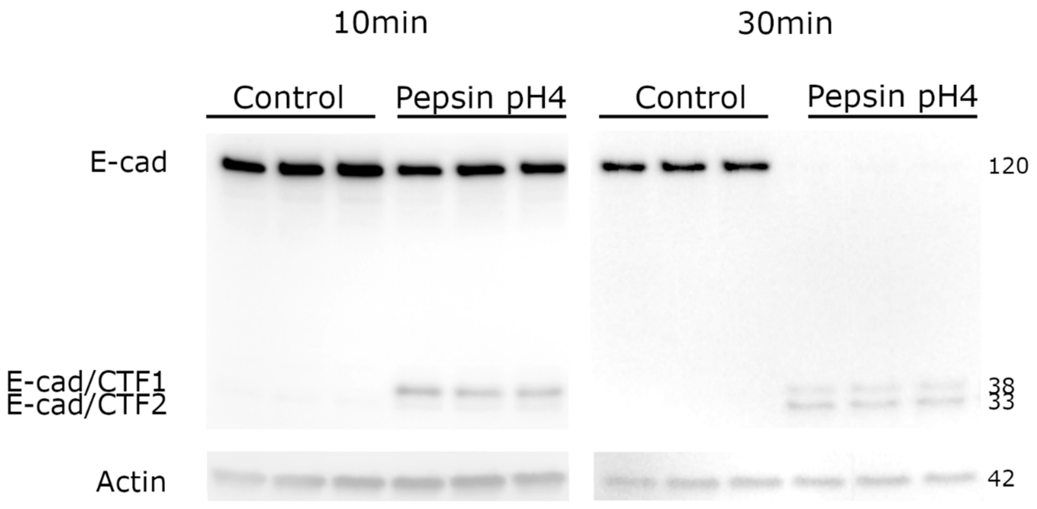

Statistical Analyses Results

I.ATP assay: Cell viability (Figure 1)

Group	N Obs	Mean	Std Dev	Minimum	Lower Quartile	Median	Upper Quartile	Maximum
**Control**	**8**	13290688.88	837162.54	11786739.00	12963682.50	13219295.50	13814795.50	14543225.00
**SA-PB + Pepsin pH4**	**8**	11379992.50	2140689.07	7189483.00	9992467.50	12346628.00	12947696.50	13276873.00
**SA-SB-CC + Pepsin pH4**	**8**	10657598.25	1839555.77	8780505.00	9060248.50	10165562.00	12340782.50	13347095.00
**Pepsin pH4**	**7**	404226.00	129900.65	296963.00	329360.00	361200.00	402957.00	686131.00
**Placebo + Pepsin pH4**	**8**	432084.13	376680.81	101289.00	133052.50	272579.00	699427.50	1145266.00

Comparison	P-value
Control vs. Pepsin pH4	<0.0001
Control vs. Placebo+Pepsin pH4	<0.0001
Control vs. SA-PB+Pepsin pH4	0.043
Control vs. SA-SB-CC+Pepsin pH4	0.0024
Pepsin pH4 vs. Placebo+Pepsin pH4	0.85
Pepsin pH4 vs. SA-PB+Pepsin pH4	<0.0001
Pepsin pH4 vs. SA-SB-CC+Pepsin pH4	<0.0001
Placebo+Pepsin pH4 vs. SA-PB+Pepsin pH4	<0.0001
Placebo+Pepsin pH4 vs. SA-SB-CC+Pepsin pH4	<0.0001
SA-PB+Pepsin pH4 vs. SA-SB-CC+Pepsin pH4	0.48

II.ATP assay: Proliferation (Figure 6)

Group	N Obs	Mean	Std Dev	Minimum	Lower Quartile	Median	Upper Quartile	Maximum
**Acid**	**24**	9657921.58	326544.85	9202465.00	9461319.50	9574875.50	9836059.00	10584557.00
**Control**	**24**	11808629.38	1688947.98	9120330.00	9645761.00	12625646.00	13000304.50	14233158.00
**Pepsin pH4**	**22**	16653334.68	1874353.27	14682413.00	15205047.00	15949018.50	19099147.00	19735751.00

Comparison	P-value
Control vs. Acid	<0.0001
Control vs. Pepsin pH4	<0.0001
Acid vs. Pepsin pH4	<0.0001

Figure 3 Replicate images

Scale bars = 50υm
Blue: DAPI
Green: E-cadherin

Control

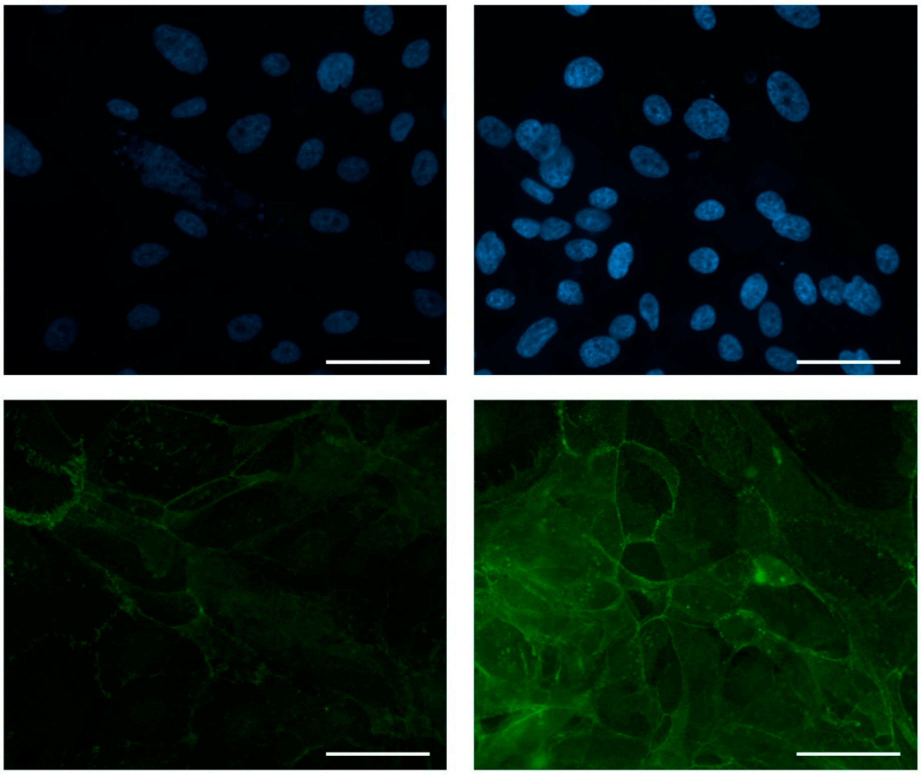

pH4

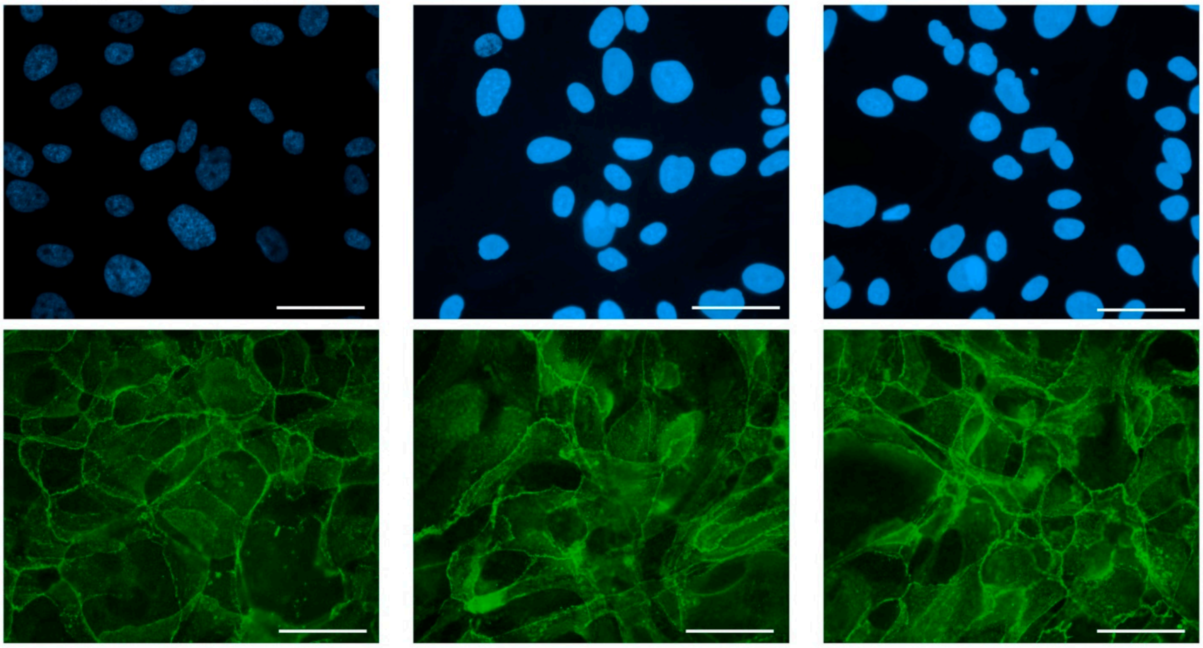

Pepsin pH4

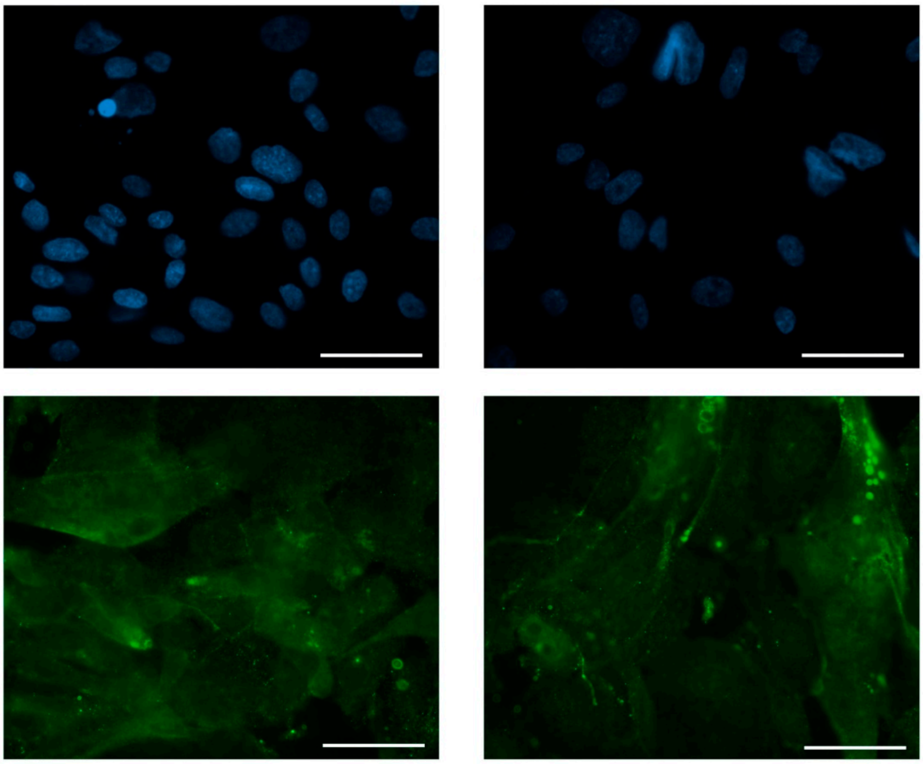

Placebo + Pepsin pH4

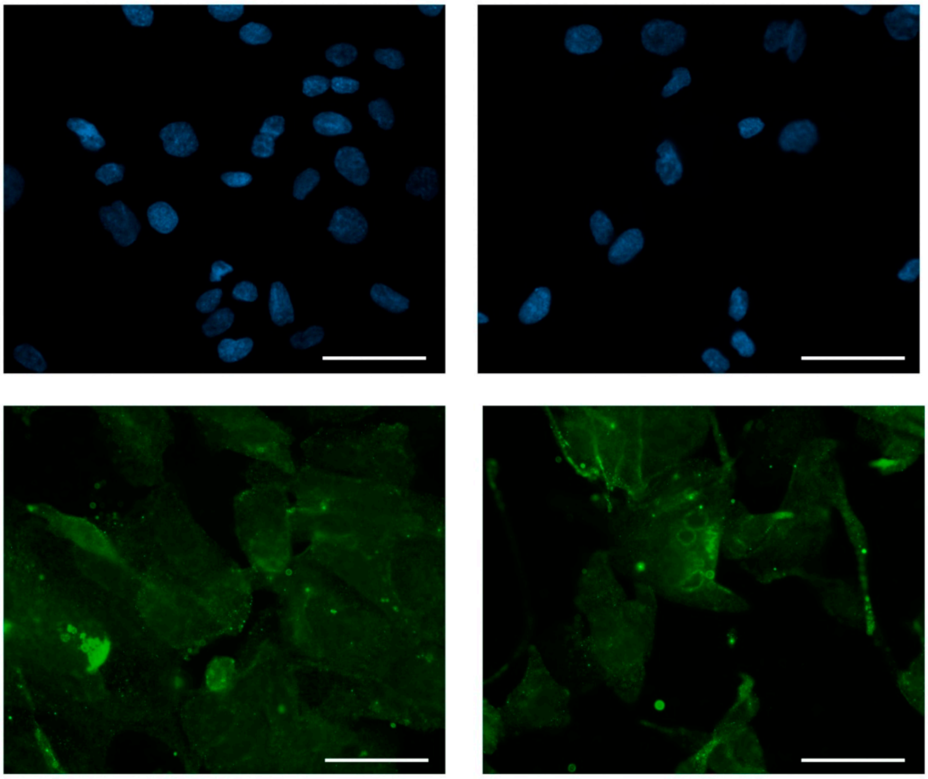

SA-PB + Pepsin pH4

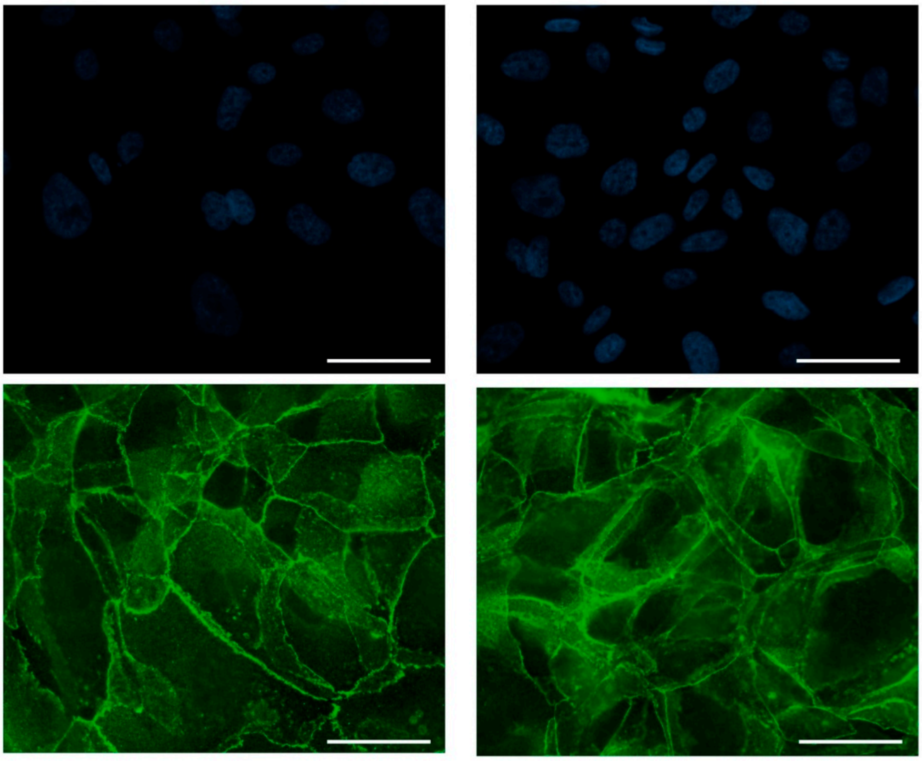

SA-SB-CC + Pepsin pH4

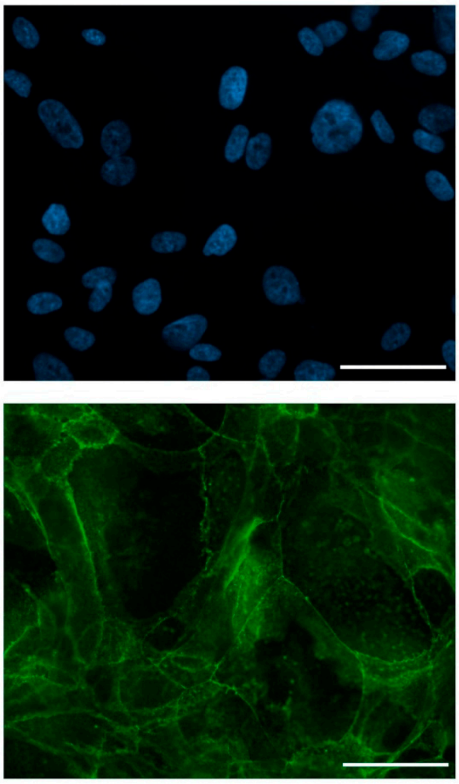

IgG

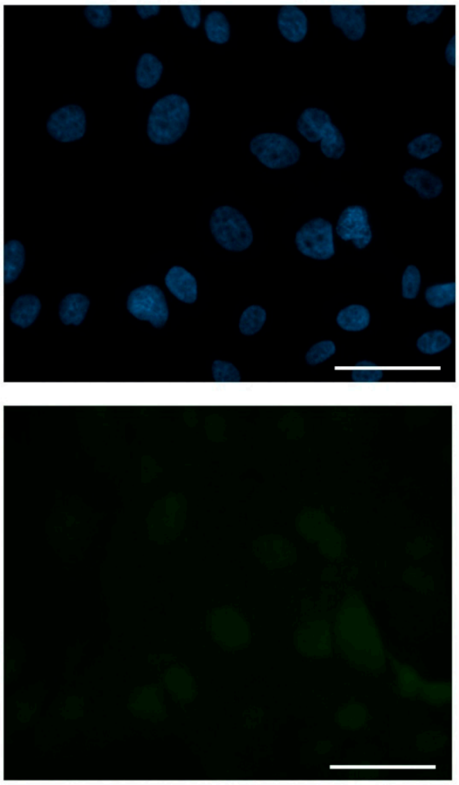

Supplement: Supplementary file 1 [file ijms-24-07932-s001.zip › ijms-2280767-supplementary.pdf]
